# Drug resistance in diploid yeast is acquired through dominant alleles, haploinsufficiency, gene duplication and aneuploidy

**DOI:** 10.1371/journal.pgen.1009800

**Published:** 2021-09-23

**Authors:** Jordan B. Barney, Dakshayini G. Chandrashekarappa, Samantha R. Soncini, Martin C. Schmidt

**Affiliations:** Department of Microbiology and Molecular Genetics, University of Pittsburgh School of Medicine, Pittsburgh, Pennsylvania, United States of America; Pacific Northwest Research Institute, UNITED STATES

## Abstract

Previous studies of adaptation to the glucose analog, 2-deoxyglucose, by *Saccharomyces cerevisiae* have utilized haploid cells. In this study, diploid cells were used in the hope of identifying the distinct genetic mechanisms used by diploid cells to acquire drug resistance. While haploid cells acquire resistance to 2-deoxyglucose primarily through recessive alleles in specific genes, diploid cells acquire resistance through dominant alleles, haploinsufficiency, gene duplication and aneuploidy. Dominant-acting, missense alleles in all three subunits of yeast AMP-activated protein kinase confer resistance to 2-deoxyglucose. Dominant-acting, nonsense alleles in the *REG1* gene, which encodes a negative regulator of AMP-activated protein kinase, confer 2-deoxyglucose resistance through haploinsufficiency. Most of the resistant strains isolated in this study achieved resistance through aneuploidy. Cells with a monosomy of chromosome 4 are resistant to 2-deoxyglucose. While this genetic strategy comes with a severe fitness cost, it has the advantage of being readily reversible when 2-deoxyglucose selection is lifted. Increased expression of the two *DOG* phosphatase genes on chromosome 8 confers resistance and was achieved through trisomies and tetrasomies of that chromosome. Finally, resistance was also mediated by increased expression of hexose transporters, achieved by duplication of a 117 kb region of chromosome 4 that included the *HXT3*, *HXT6* and *HXT7* genes. The frequent use of aneuploidy as a genetic strategy for drug resistance in diploid yeast and human tumors may be in part due to its potential for reversibility when selection pressure shifts.

## Author summary

Yeast and fast-growing human tumor cells share metabolic similarities in that both cells use fermentation of glucose for energy and both are highly sensitive to the glucose analog 2-deoxyglucose. In an earlier study, we examined the genetic mechanism used by haploid yeast cells to acquire resistance to 2-deoxyglucose. Here, we conducted the same genetic selection on diploid cells. Diploid cells use dominant alleles, haploinsufficiency, gene duplication and aneuploidy to achieve drug resistance. The frequent use of aneuploidy as a genetic strategy for drug resistance in diploid yeast and human tumors may be in part due to its potential for reversibility when selection pressure shifts.

## Introduction

The yeast *Saccharomyces cerevisiae* has been used as a model eukaryotic organism for genetic studies due to its many properties that are advantageous to laboratory research. These advantages include its short generation time, a small and well-annotated genome, robust recombination systems and its ability to stably grow in either haploid or diploid states [1]. Most genetic screens have utilized haploid strains since this allows phenotypic expression of recessive alleles. In contrast, genetic screens in diploids have been much less commonly conducted for several reasons. First, recessive alleles are often phenotypically silent. Second, the isolation and identification of dominant mutations is cumbersome and often required production of individual libraries specific for each mutation. The advent of next generation sequencing circumvents this second challenge to using diploid cells and provides a faster and easier method for identification of genetic changes in diploid strains.

Genetic studies of the yeast cell’s adaptation and response to the metabolic inhibitor 2-deoxyglucose (2DG) have identified many of the genes required for the maintenance of and release from glucose repression [2–5]. All of these studies were conducted in haploid cells growing on fermentable carbon sources other than glucose. We recently conducted a screen for spontaneous mutations that conferred 2DG resistance in haploid cells growing on glucose [6] Here, we report the identical selection methodology used in our earlier report but use diploid cells instead. We wanted to define the different genetic mechanisms used in haploid versus diploid cells in response to the same metabolic challenge. At the outset, we expected that a genetic screen for 2DG resistance in haploid cells would uncover recessive alleles while a similar screen in diploids would uncover dominant alleles. While this expectation was realized, in diploid cells a second and more prevalent genetic strategy to respond to this metabolic challenge was to change chromosome numbers. We observed that increased (trisomies and tetrasomies) and decreased (monosomies) chromosome numbers served as an effective and reversible genetic strategy for response to the presence of 2DG. Additional mechanisms to alter gene expression include haploinsufficiency to reduce expression of *REG1* and duplication of a large segment on chromosome 4 that increased expression of three hexose transporters genes. While many of the genetic mechanisms for the acquisition of 2DG-resistance were utilized by both haploid and diploid cells, the use of a monosomy and haploinsufficiency are options only possible in diploid cells. As has been suggested for other traits in yeast [7–9], these studies highlight the utility and potential for reversibility of aneuploidy as a genetic strategy that pertains to both diploid yeast in response to 2DG and human tumors cells in response to therapeutics and other selective pressures during the course of tumor development.

## Results

### Isolation of spontaneous 2DG-resistant mutants in diploid cells

In order to obtain spontaneous mutants with resistance to 2DG, multiple, independent colonies of diploid yeast (MSY1527; Table 1) were grown overnight in liquid cultures of synthetic complete medium with 2% glucose. The following day, approximately 10^7^ cells were spread onto agar plates containing synthetic complete media with 2% glucose and supplemented with 0.1% 2DG. Plates were incubated for 4–6 days until colonies appeared. The frequency at which spontaneous 2DG-resistant colonies appeared was approximately 4–6 colonies per 10^7^ cells. These 2DG-resistant candidates were propagated in synthetic complete medium, and cultures were preserved in glycerol stocks at -80°C. Initial characterization of the 2DG-resistant isolates was conducted by examining growth properties of the candidates on agar plates in the absence or presence of 0.1% 2DG. Candidates which failed to retain significant resistance to 2DG were discarded. Nine of the isolates exhibited normal growth properties and stable resistance to 2DG. Seven of the 2DG-resistant diploid isolates formed small colonies when grown on agar plates lacking 2DG (Table 2) and showed a high frequency of reversion to a state that was 2DG sensitive and with normal colony size (discussed in detail below).

**Table 1:** Yeast strains.

Strain	Genotype
MSY1333	*MATα ura3 leu2 his3 HXT3-GFP*::*His3MX*
MSY188	*MAT* ***a*** *ura3-52 his3Δ200*
MSY189	*MATα ura3-52 leu2Δ1*
MSY1527	Diploid MSY1333 x MSY188
MSY1212	*MAT* ***a*** *ura3-52 leu2Δ1 his3Δ200*
MSY520	*MAT****a****ura3-52 leu2Δ1 trp1Δ63 his3Δ200 sip2Δ*::*HIS3*
MSY522	*MAT****a****ura3-52 leu2Δ1 trp1Δ63 his3Δ200 gal83Δ*::*HIS3*
MSY534	*MATα ura3-52 leu2Δ1 trp1Δ63 his3Δ200 sip1Δ*::*HIS3*
MSY850	*MAT* ***a*** *ura3-52 leu2Δ1 his3Δ200 snf4Δ1*
MSY1313	*MAT* ***a*** *ura3-52 leu2Δ1 his3Δ200 snf1Δ10*
MSY930	*MAT****a****ura3Δ0 leu2Δ0 his3Δ1 met15Δ0 reg1Δ*::*KAN*
MSY1520	*MATα ura3 leu2 his3 HXT3-GFP*::*His3MX reg1-Q332Stop*
MSY841	*MATα ura3 leu2 lys2Δ0 trp1Δ63 reg1Δ*::*URA3*
MSY1553	*MAT* ***a*** *ura3-52 his3Δ200 disomy Chr 8*
MSY1556	*MAT****a****/α ura3/ura3 leu2/LEU2 his3/his3 HXT3/ HXT3-GFP*::*His3MX tetrasomy Chr 8*
MSY1564	*MATα ura3-52 his3Δ1 +* duplication of *HXT367* cluster on Chr 4
MSY1558	Diploid 1553 x 189; trisomy Chr 8
MSY1560	*MAT****a****ura3-52 his3Δ200 +* disomy Chr 8 *dog1/2Δ*::*HIS3*
MSY1561	*MAT****a****ura3-52 his3Δ200 sip2Δ*::*HIS3 +* disomy Chr 8
MSY1562	*MAT****a****ura3-52 his3Δ200 +* disomy Chr 8 *hxt415Δ*::*HIS3*

**Table 2:** Genomic Sequencing Results.

Isolate Name	Candidate Mutations	Aneuploid Chromosomes			Growth property
		Monosomy	Trisomy	Tetrasomy	
DS2	none		8,11,12,14,15	13	normal
DS5	none		14	8	normal
DS6	none		3,5,8,11,12,14		normal
DS7	*REG1-Y638Stop*		8		normal
DS14	none	1		8	normal
DS18	none		5,9,10,11,13,14,16	3,8	normal
DS15	*REG1-Q332Stop*				normal
DS16	*GAL83-S224R*				normal
DS21	*SNF4-N177S*				normal
DS8	none	4			slow growth
DS9	duplication of 117 kb region (3 *HXT* genes)	4	2		slow growth
DS10	none	4			slow growth
DS11	none	4			slow growth
DS12	none	4			slow growth
DS13	none	4			slow growth
DS19	none	4			slow growth

### Whole genome sequencing of 2DG-resistant strains

**Fig 1:**
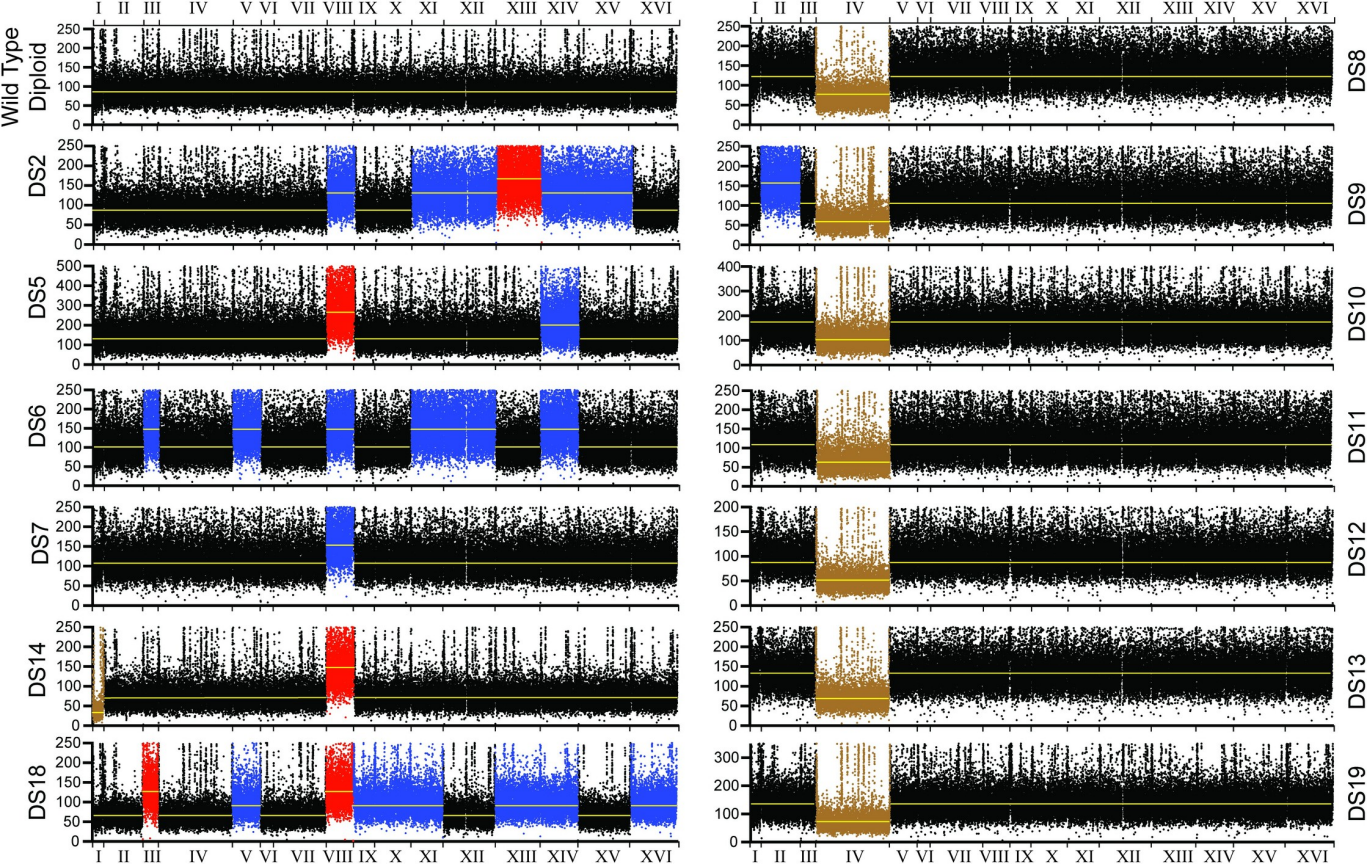
Aneuploidy in 2DG-resistant diploid strains. Diploid strains were sequenced, and the read depth (y-axis) is plotted against chromosomal position (x-axis) with chromosome number shown in Roman numerals. Median read depth is indicated with a yellow line. Monosomic chromosomes are plotted in brown, trisomic chromosomes in blue and tetrasomic chromosomes in red.

In order to identify the genetic changes that mediated 2DG resistance, diploid strains were subjected to whole genome sequencing using the Illumina platform to produce 151-bp paired-end reads. Typical sequencing reactions produced 10–20 million reads, sufficient for ~100-fold coverage across the yeast genome. Reads were mapped to the S288c genome scaffold with Bowtie2 and analyzed for sequence variants that could be considered as candidates for mutations that confer 2DG resistance. In addition, changes in chromosome ploidy were examined using BEDTools to measure read depth along each chromosome. A plot of read depth versus chromosomal position revealed that 13 of the 16 2DG-resistant isolates were aneuploid (Fig 1). Chromosomes with twice the normalized median read depth were considered to be tetrasomic, those with half the median read depth represented monosomies and those 1.5 times the normalized median read depth represented trisomies. The aneuploid strains could be divided into two distinct groups, those with extra copies of chromosome 8 (trisomies in DS2, DS6 and DS7; tetrasomies in DS5, DS14 and DS18) and those with a monosomy of chromosome 4. The seven strains that showed the slow growth phenotype all contained a monosomy of chromosome 4. The three strains without aneuploidy contained sequence variants that were strong candidates for mutations responsible for 2DG resistance (Table 2). Two strains (DS7 and DS15) contained stop codons in the *REG1* gene. Two strains contained missense mutations in the beta and gamma subunits (*GAL83-S224R* and *SNF4-N177S*, *respectively*) of yeast AMP-activated protein kinase (AMPK, also known as the Snf1 kinase). One strain (DS9) contained both an aneuploid state (monosomy of chromosome 4 and trisomy of chromosome 2) as well as a 117 kb duplication of a region of the chromosome 4 (barely visible in Fig 1 but discussed in more detail below).

### Dominant mutations in genes encoding all three subunits of yeast AMPK confer 2DG resistance

**Fig 2:**
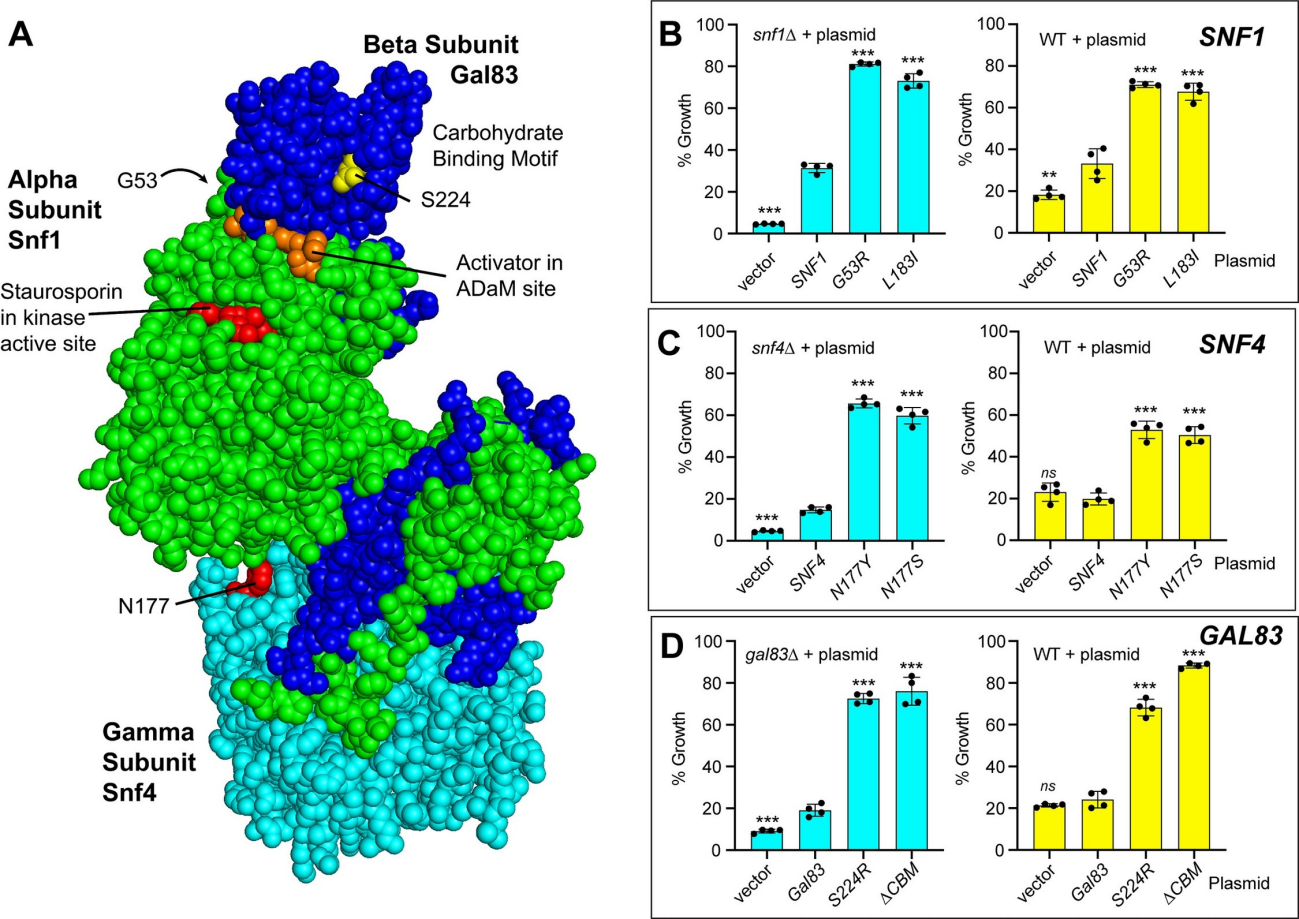
Dominant alleles in the Snf1 kinase complex confer 2DG resistance. **A**. The structure the mammalian AMPK (6c9h.pdb) bound to the activator R734 [46] is shown as a model for the Snf1 kinase heterotrimer. The alpha subunit is shown in green, the beta subunit in blue and the gamma subunit in cyan. The locations of the kinase domain active site, the allosteric drug and metabolite-binding site (ADaM) and the dominant mutations that confer 2DG resistance are shown. **B-D.** 2DG resistance assays performed in quadruplicate using wild type (WT) and gene deletion strains transformed with the indicated plasmids that express a wild type gene, the same gene with a single missense mutation indicated or empty plasmid vector. Mean values statistically different from cells expressing the wild type allele are indicated.

By selecting for 2DG resistance in diploid cells, we expected to recover dominant alleles. In our earlier screen with haploid cells, we identified one dominant allele that could confer 2DG resistance and that mapped to alpha subunit of AMPK, the *SNF1* gene. In this study we identified missense alleles in the gamma subunit gene (*SNF4)* in DS21 and in the beta subunit gene (*GAL83)* in DS16 (Table 2). In order to test whether these amino acid substitutions were sufficient to confer 2DG resistance, the substitutions were introduced into low-copy plasmids and reintroduced into naïve yeast strains (strains not previously exposed to 2DG) with a deletion in the gene of interest. To test for dominance, the plasmids were also introduced into naïve wild type strains (Fig 2). For comparison, we also performed the same analysis using two dominant alleles of *SNF1*, *SNF1-G53R* [6] and *SNF1-L183I* [10]. When 2DG resistance was measured in quadruplicate in the *snf1Δ* strain, we detected 2DG hypersensitivity in cells transformed with plasmid vector and significant 2DG resistance when expressing Snf1-G53R or Snf1-L183I (Fig 2B), findings consistent with our earlier studies [6,11]. In wild type cells, we found that increased gene dosage of wild type *SNF1* on a low-copy plasmid conferred modest but significant 2DG resistance and that the *SNF1-G53R* and *SNF1-L183I* alleles both exhibited dominant and robust 2DG-resistance.

Using the same assay system, we analyzed the *SNF4-N177S* allele for 2DG resistance and dominance. We included another allele of *SNF4* that was identified previously as a dominant-acting regulator of *INO1* transcription that contained different amino acid substitution at the same asparagine residue, *SNF4-N177Y* [12]. We found that cells lacking *SNF4* were hypersensitive to 2DG (Fig 2C), a result consistent with the requirement of the gamma subunit for full function of the AMPK heterotrimer [13,14]. Both the *SNF4-N177S* and *SNF4-N177Y* alleles conferred 2DG resistance to *snf4Δ* and the *SNF4*^*+*^ cells, confirming that these are dominant-acting alleles of *SNF4*. The gamma subunit of AMPK is known to bind directly to adenylate nucleotides [15] and to transduce adenylate charge information to the Snf1 kinase [16]. The N177 residue is not in proximity to the adenylate binding pockets (Fig 2A) and its effect on the regulation of the Snf1 kinase is not apparent from the structure of the heterotrimer.

In addition to dominant alleles of the *SNF1* and *SNF4* loci, we also identified a novel missense allele in the *GAL83* gene which encodes one of the three alternative beta subunits of the AMPK heterotrimer [17]. To characterize *GAL83-S224R* allele, the amino acid change was engineered into a low-copy *GAL83* plasmid and introduced into wild type and *gal83Δ* cells. The location of this amino acid is within a domain now known as the carbohydrate binding module (CBM) and previously as the glycogen binding domain (GBD). This domain is conserved among all AMPK beta subunits from yeast to mammals [18]. To further the analysis of this allele, the corresponding missense alleles were also constructed in the *SIP1* and *SIP2* genes (S1 Fig) which encode the other AMPK beta subunits in yeast. Finally, we included in this analysis beta subunit genes with complete deletions of the CBM domain since we had earlier determined that the *GAL83-ΔCBM* gene was a dominant allele that could confer 2DG resistance [19]. 2DG resistance assays showed that changes in the *GAL83* locus had strong effects on cell growth. Deletion of *GAL83* resulted in increased sensitivity to 2DG similar to that observed in *snf1Δ* and *snf4Δ* cells (Fig 2D). Both the *GAL83-S224R* and the *GAL83-ΔCBM* alleles conferred significant resistance to 2DG and did so in a dominant manner. A very different response to these analogous mutations was observed for the *SIP1* and *SIP2* genes (S1C and S1D Fig). Deletion of these genes had no significant effect on 2DG sensitivity. Neither the *SIP2-S226R* nor the *SIP1-S541R* allele was able to confer any significant 2DG resistance while deletion of the CBM domain in *SIP2* was able to confer dominant 2DG resistance. Thus, the three AMPK isoforms of yeast are distinguished by the identity of their beta subunit with each showing a distinct regulation profile based on amino acid changes in or deletion of the CBM.

### Haploinsufficiency of the REG1 locus confers 2DG resistance

**Fig 3:**
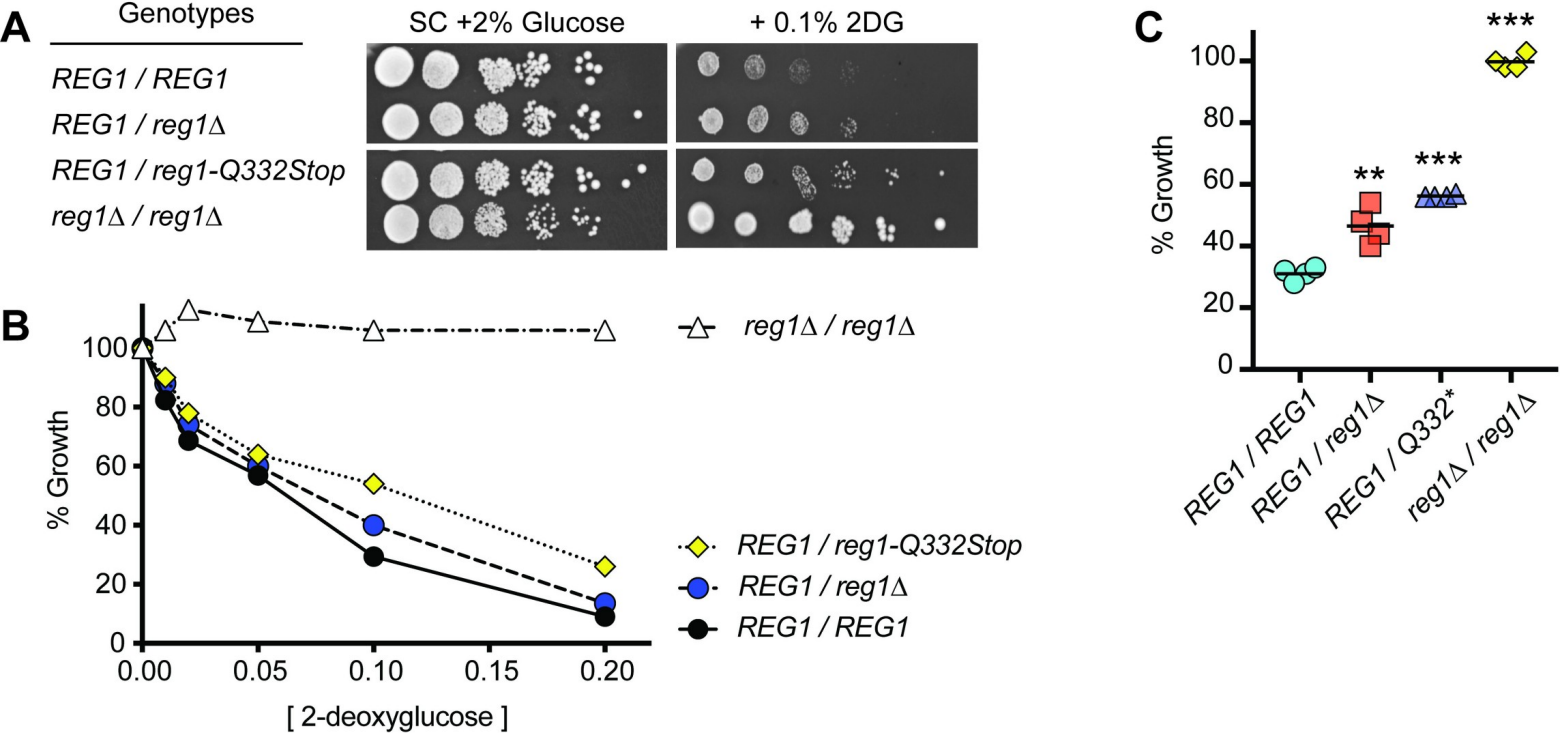
Haploinsufficiency of *REG1* confers 2DG resistance. **A.** Diploid cells with the indicated genotypes at the *REG1* locus were serially diluted and spotted onto agar plates containing SC medium with 2% glucose with and without 0.1% 2DG. **B.** 2DG resistance assay of diploid cells with the indicated genotypes. **C.** 2DG resistance measured in quadruplicate using 0.1% 2DG. Diploid strains used in this experiment were as follows: *REG1/REG1* (MSY1333 x MSY188); *reg1Δ/REG1* (MSY1333 x MSY930); *reg1-Q332Stop/REG1* (MSY188 x MSY1520); *reg1Δ/reg1Δ* (MSY930 x MSY841).

Two of the 2DG resistant diploid strains were heterozygous at the *REG1* locus owing to stop codons in the open reading frame in one copy (Table 2). In DS15, one *REG1* allele contained a stop codon in position 332 while DS7 had a stop codon at position 638. Previous studies of C-terminal deletions in the Reg1 protein suggested that stop codons at these positions would generate loss-of-function alleles [20]. To test whether cells heterozygous for *REG1* were 2DG-resistant, we generated a series of diploid strains that varied only at the *REG1* locus and measured 2DG resistance using spot dilution assays (Fig 3A) and 2DG titration assays (Fig 3B). Cells heterozygous at the *REG1* locus manifested modest 2DG resistance that was reproducible and statistically significant (*p*<0.01; Fig 3C). Homozygous *reg1* cells (*reg1Δ/reg1Δ*) demonstrate even greater 2DG resistance but this comes with a heavy fitness cost [6] that is not observed in the heterozygous cells. These data demonstrate that haploinsufficiency at the *REG1* locus confers 2DG resistance.

### Monosomy of chromosome 4 confers 2DG resistance

**Fig 4:**
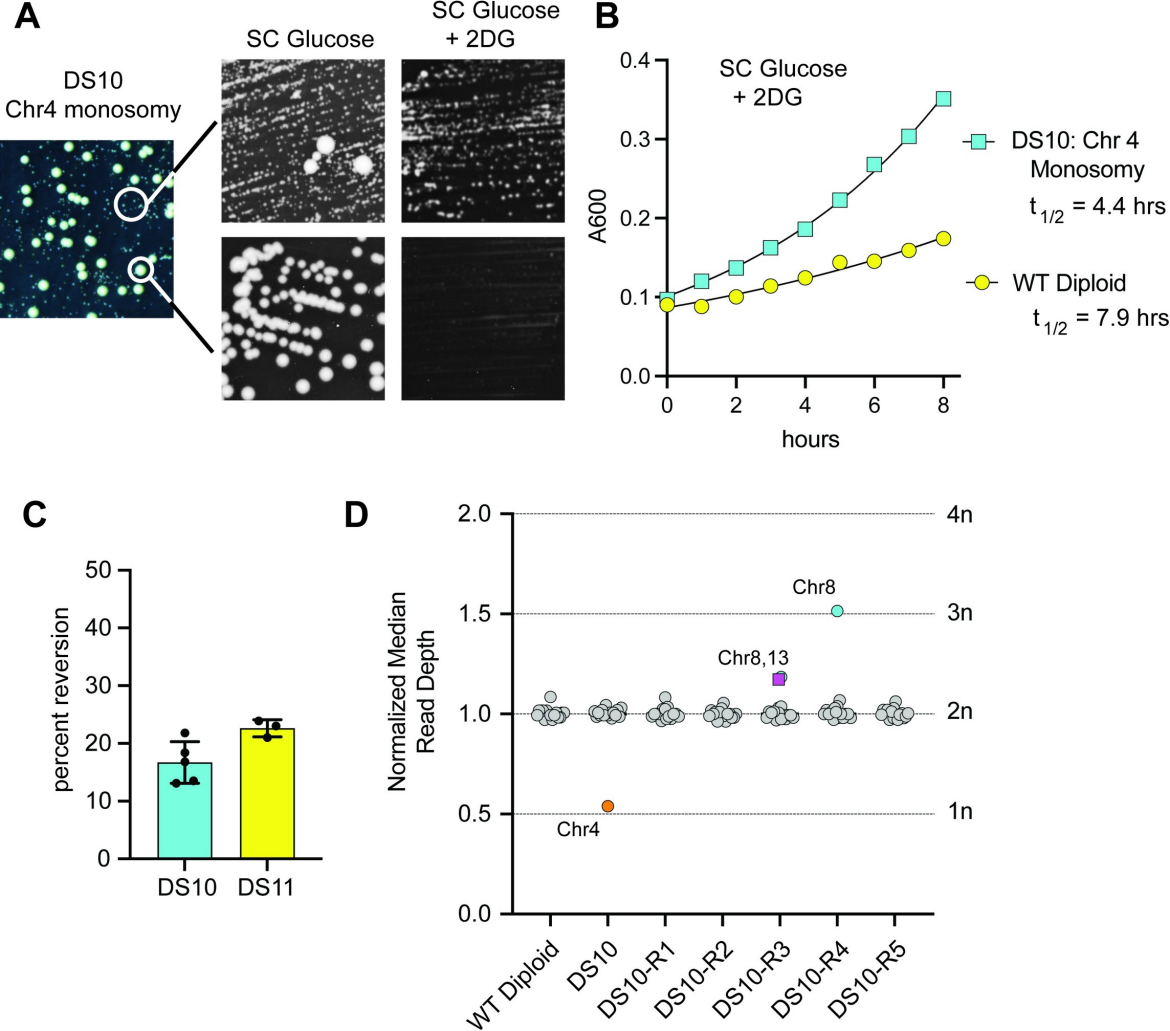
Monosomy of chromosome 4 confers 2DG resistance and reverts at high frequency. **A.** Growth properties DS10 cells on agar plates. Small and large colonies of DS10 were re-streaked onto agar plates with the media indicated above. **B.** Growth of DS10 cells and wild type diploid cells in media containing 2DG. **C**. Reversion of small colony phenotype. Multiple, independent populations of small colonies from strains DS10 and DS11 were spread on plates containing SC medium. The mean percentage (±SD) of colonies that reverted to the large colony phenotype for each independent replicate is shown. **D.** Chromosomal ploidy analysis of DS10 and revertants. Normalized median read depth for each chromosome is plotted for wild type, DS10 and five DS10 revertants (DS10-R). Chromosomes that diverge from normal ploidy (2n) are indicated.

Seven of the 2DG-resistant diploid strains contained a monosomy of chromosome 4 (Fig 1). These isolates all shared the unusual growth characteristic of presenting one of two distinct phenotypes when grown on agar plates: small, slow-growing colonies and large, fast-growing colonies. To characterize these isolates, the small colonies and large colonies were picked and streaked onto agar plates with and without 2DG (Fig 4A). The large colonies gave rise only to large colonies and were not resistant to 2DG. In contrast, the small colonies were not stable since they gave rise to both small and large colonies when grown in the absence of 2DG. Only the small colonies exhibited 2DG resistance with a shorter doubling time when grown in liquid media with 2DG (Fig 4B). Working with the 2DG-resistant, small colonies proved to be challenging since they could not be grown in liquid media without 2DG selection since the large colony-forming cells arise at high frequency and take over the culture. The only way to obtain a relatively pure population of small colony, 2DG-resistant cells was to wash them off agar plates after physically removing the large colonies. When diplopid cells monosomic for chromosome 4 were allowed to form colonies in the absence of 2DG selection, we found that approximately one in every 5 colonies had reverted to the large colony phenotype (Fig 4C). We hypothesized that the small colony-forming, 2DG-resistant cells contained the chromosome 4 monosomy and that the large colony-forming, 2DG-sensitive cells were revertants with a restored 2n ploidy of chromosome 4. To test this, we washed the small colonies off an agar plate and subjected them to whole genome sequencing. For comparison, we also sequenced the wild type diploid and 5 independent, revertants (large colonies). Ploidy analysis using normalized median read depth for each chromosome showed that the small colony-forming, 2DG-resistant cells did contain a monosomy of chromosome 4 while the revertants all restored normal 2n ploidy of chromosome 4 (Fig 4D). We also found that 2 of the 5 revertants exhibited novel aneuploid states with extra chromosomes that arose during the process of restoring 2n ploidy of chromosome 4. We interpret the two chromosomes in DS10-R3 with increased ploidy between 2n and 3n as being a mixed population with some cells having normal 2n ploidy and some having trisomies of chromosomes 8 and 13. In summary, these data demonstrate the following: 1) cells with a monosomy of chromosome 4 are 2DG-resistant; 2) in the absence of 2-deoxyglucose, this aneuploid state comes with a high fitness cost as judged by slow growth and small colony phenotype; 3) monosomy of chromosome 4 is readily reversible and 4) reversion of the chromosome 4 monosomy to normal ploidy is often accompanied by aberrant segregation of other chromosomes.

### 2DG resistance conferred by extra copies of chromosome 8

Examination of the diploid strains exhibiting aneuploidy revealed that six of the 2DG resistant strains (DS2, DS5, DS6, DS7, DS14, DS18) shared the trait of having extra copies of chromosome 8 (Fig 1). In an earlier selection for 2DG resistance using haploid cells, we isolated three aneuploid strains with disomies of multiple chromosomes including, in each case, a disomy of chromosome 8 [6]. In order to determine which of the disomic chromosome (s) were important for 2DG-resistance, we mated three independent aneuploid strains containing multiple disomic chromosomes with a wild type strain and subjected the diploids to sporulation. The haploid progeny were assayed for 2DG resistance and 4 tetrads from each cross were chosen based on the 2:2 segregation of 2DG resistance (S2 Fig). DNA extracted from pools of the eight 2DG-resistant and the eight 2DG-sensitive strains were analyzed by whole genome sequencing. The chromosomal ploidy change that best explained the 2DG resistance was the disomy of chromosome 8. Our data were consistent with the idea that extra copies of chromosome 8 were sufficient for 2DG resistance and that the other disomic chromosomes did not significantly contribute to 2DG-resistance.

**Fig 5:**
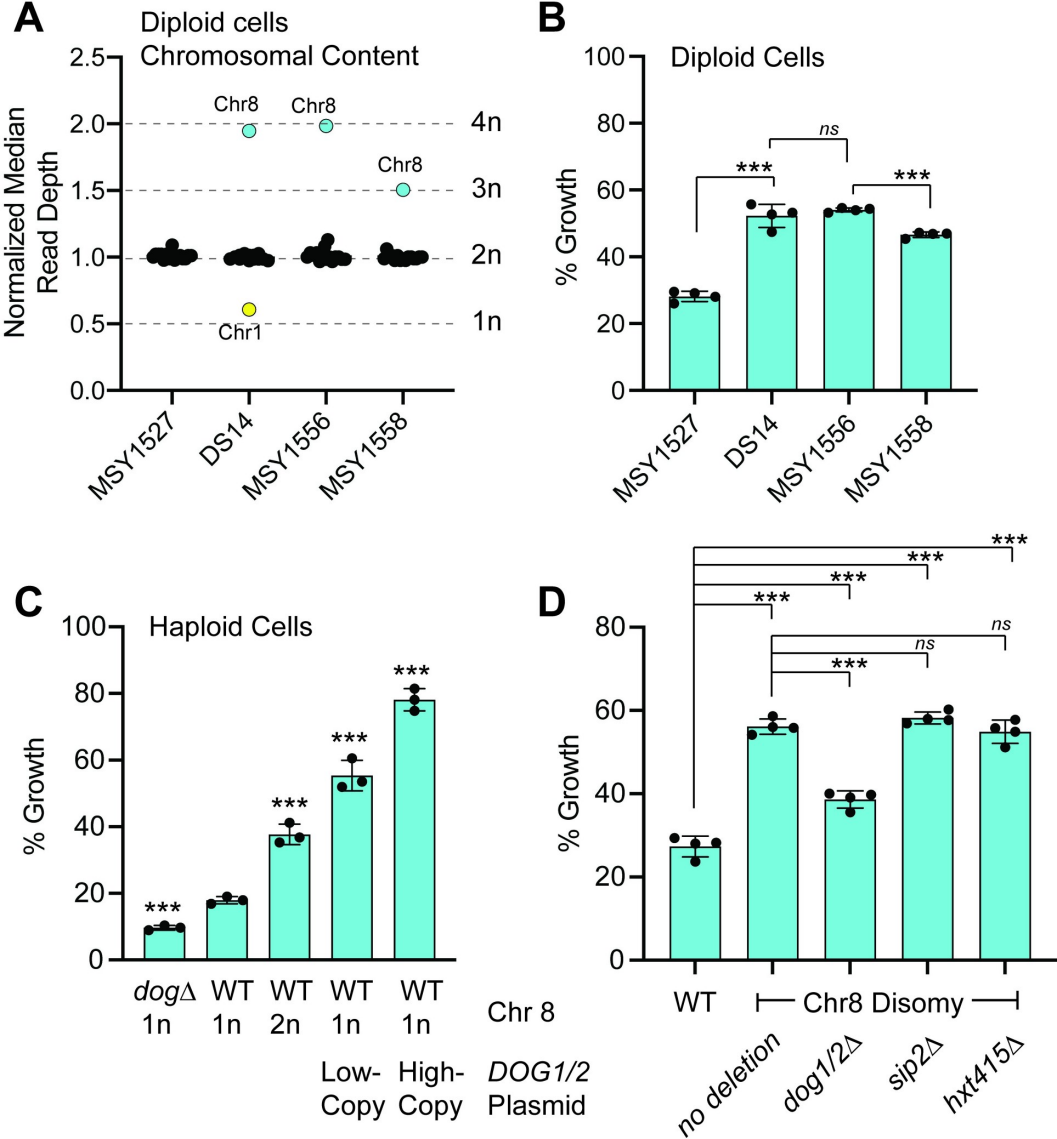
Increased copy number of *DOG*1/2 genes mediates 2DG resistance. **A.** Normalized median read depth of chromosomes in wild type diploid strain (MSY1527), in the 2DG-resistant isolate, DS14, and in diploids generated from the haploid progeny of DS14. Chromosomes with 2n copy number are shown in black. Aneuploid chromosomes are indicated. **B.** 2DG resistance assay of diploid strains shown in A. **C.** 2DG resistance was measured in haploid strains with increasing copy number of the *DOG1/2* genes. Resistance was compared to wild type (WT) haploid cells with 1n ploidy of chromosome 8 and mean values statistically different are indicated. **D.** 2DG resistance assays of a wild type haploid strain and haploid strains with a disomy of chromosome 8 were performed in quadruplicate. Deletions were engineered in the disomic strains to remove the *DOG1/2* or *HXT415* genes from one of the disomic chromosomes or the *SIP2* gene on chromosome 7.

Earlier studies have found that increased expression of two closely related phosphatases, Dog1 and Dog2, confers resistance to 2DG [21,22]. The *DOG1* and *DOG2* genes are adjacent to each other and are located on chromosome 8. We next analyzed the diploid strain DS14 which contained a chromosome 1 monosomy and chromosome 8 tetrasomy. DS14 was subjected to sporulation and the viable progeny (haploid spores lacking chromosome 1 are not viable) were sequenced and used to generate diploid strains with 2, 3 or 4 copies of chromosome 8. Chromosome copy number was confirmed by whole genome sequencing and comparison of the normalized median read depth for each chromosome (Fig 5A). When each strain was subjected to 2DG-resistance assays in quadruplicate, the original isolate DS14 was significantly resistant to 2DG when compared to the reference diploid strain MSY1527 (Fig 5B). Cells with a tetrasomy of chromosome 8 (MSY1556) showed 2DG resistance indistinguishable from the DS14 thus demonstrating that the tetrasomy of chromosome 8 was sufficient for 2DG resistance and that the chromosome 1 monosomy did not confer any additional 2DG resistance. Resistance conferred by chromosome 8 was dose-dependent since the strain with chromosome 8 trisomy exhibited intermediate resistance compared to a wild type diploid (*2n*) and the strain with chromosome 8 tetrasomy. The dose dependence of *DOG* gene copy number and 2DG resistance was further confirmed with haploid strains with 0, 1, 2 or more copies of the *DOG1* and *DOG2* genes (Fig 5C).

To confirm that the *DOG* genes were necessary for chromosome 8 aneuploid mediated 2DG resistance, we generated a set of haploid strains each with a chromosome 8 disomy that were deleted for specific genes. Homologous recombination was used to replace the *DOG1* and *DOG2* locus on one of disomic chromosome 8’s with the *HIS3* gene to generate the *dog1/2Δ*::*HIS3* allele. A second possible mechanism for 2DG resistance in cells with extra copies of chromosome 8 is the increased expression of the three glucose transporters (*HXT4*, *HXT1* and *HXT5*) also located on this chromosome. Increased expression of *HXT1* and plasma membrane retention [23] had been previously shown to confer 2DG resistance. Homologous recombination was used to replace the adjacent *HXT4*, *HXT1* and *HXT5* genes on one copy of the disomic chromosome 8 with the *HIS3* gene to generate the *hxt415Δ*::*HIS3* allele. Finally, as a negative control we replaced the *SIP2* gene on chromosome 7 (*sip2Δ*::*HIS3*) since we knew from earlier studies that this gene deletion did not confer 2DG resistance or sensitivity [11]. The disomy of chromosome 8 and the gene knockouts on one of the two disomic chromosomes were all confirmed by whole genome sequencing (S3 Fig). The 2DG resistance in these strains was assayed in quadruplicate (Fig 5D). Haploid cells with a disomy of chromosome 8 were significantly more resistant to 2DG when compared to a haploid (*1n*) reference strain. Deletion of the *DOG* loci from one of the disomic chromosomes significantly reduced the 2DG resistance while deletion of the *HXT* cluster and the *SIP2* gene had no effect on 2DG resistance. Thus, the extra copies of the *DOG1* and *DOG2* genes are necessary for the 2DG resistance conferred by the disomy of chromosome 8.

### Duplication of HXT genes confers 2DG resistance

**Fig 6:**
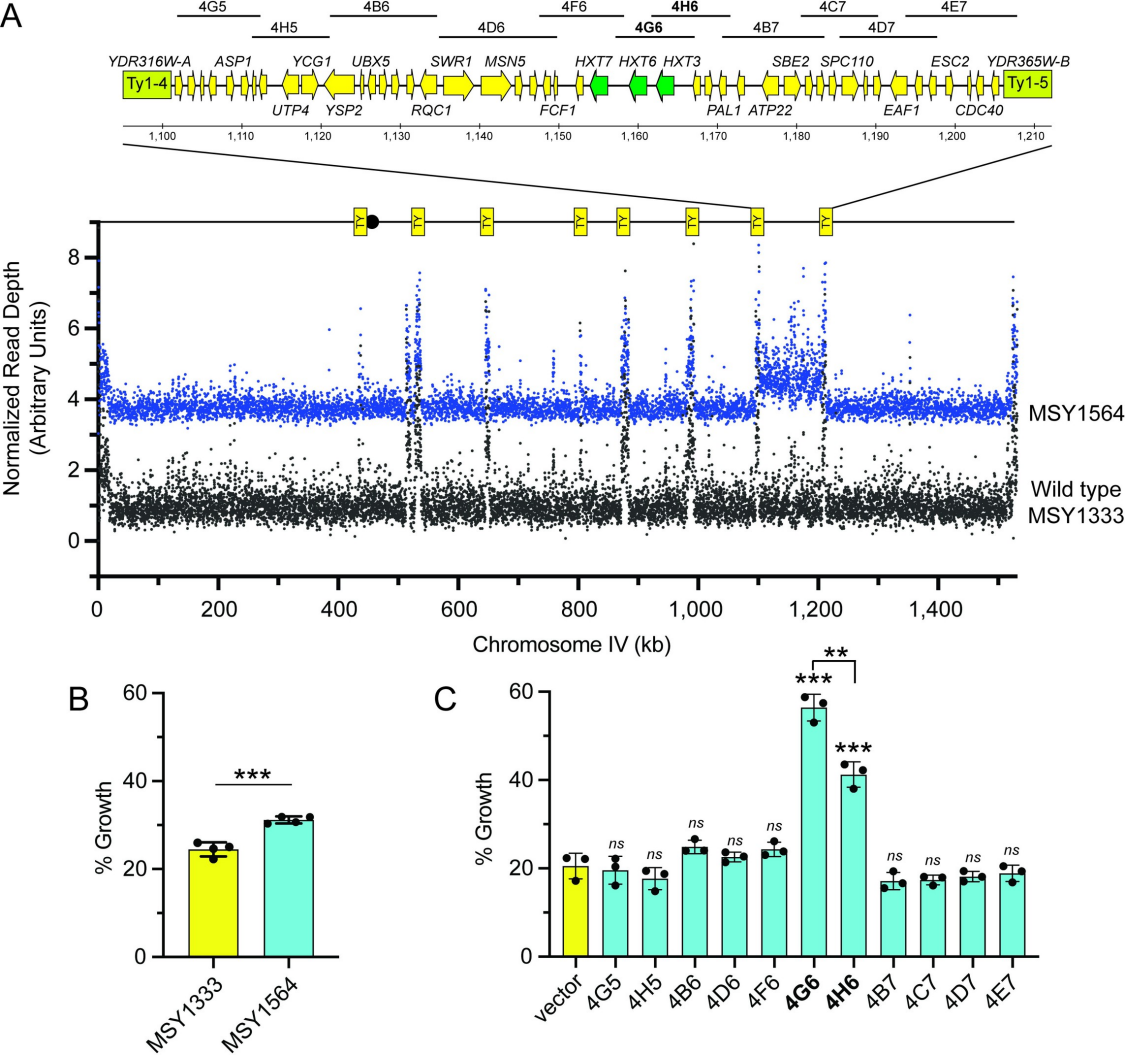
Duplication of *HXT367* cluster on chromosome 4 confers 2DG resistance. **A.** Read depth across chromosome 4 was plotted for the wild type strain (MSY1333) and the haploid strain (MSY1564) bearing the 117 kb duplicated region. Positions of Ty elements flanking the duplicated region and some of the genes within are shown. Regions present in high-copy number plasmid clones [24] covering this region are shown at top. **B.** 2DG resistance assay performed in quadruplicate showing mean ±SD and statistical significance for haploid strains (1n) with (MSY1564) and without (MSY1333) the 117 kb duplication. **C.** 2DG resistance in a wild type haploid strain transformed with high-copy number plasmids spanning the duplicated region. Statistical significance for each sample compared to vector is indicated as is a comparison of 4G6 and 4H6.

While most of our 2DG resistant strains contained a single alteration that was sufficient to confer 2DG resistance, two strains contained more than one genetic change, each capable of contributing to a 2DG-resistant phenotype. For instance, DS7 contains both a stop codon in the *REG1* gene, which can by itself confer 2DG resistance via haploinsufficiency (Fig 3), and a trisomy of chromosome 8 which confers 2DG resistance via increased gene dosage of the *DOG1/2* genes (Fig 5). Closer examination of the WGS data from DS9 revealed that a duplication of 117 kb on the monosomic chromosome 4 had occurred (Fig 6A). Sporulation of DS9 yields 2 viable haploid progeny per meiotic tetrad since loss of chromosome 4 is lethal. We were not able to recover viable haploid progeny with a disomy of chromosome 2 and could not assess its contribution to 2DG resistance. However, examination of the chromosome 4 in the viable progeny from DS9 sporulation showed that the 117 kb duplication was stably inherited. The duplication spans from the Ty1-4 element at nucleotide 1,095,767 to the Ty1-5 at nucleotide 1,212,621 and includes 52 annotated genes. Cells with this duplication exhibited modest but reproducible (*p*<0.001) 2DG resistance (Fig 6B). The genes present in this duplication that were most likely to confer 2DG resistance were the cluster of three adjacent *HXT* genes, *HXT3*, *HXT6* and *HXT7*. Earlier studies have shown that high copy plasmids encoding *HXT1* and *HXT3* can suppress the hypersensitivity to 2DG observed in *snf1Δ* cells and can confer modest resistance to wild type cells [23]. Wild type haploid cells were transformed with a set of high-copy number plasmids [24] whose inserts cover the duplicated region (Fig 6A). Three independent transformants for each plasmid was assayed for 2DG resistance. Plasmids that contained the *HXT3* gene (4G6 and 4H6) exhibited significant 2DG resistance (Fig 6C). Increased gene dosage of the *HXT7* gene did not by itself confer any significant 2DG resistance (plasmid 4F6). However, cells transformed with plasmid 4G6 containing *HXT3* and *HXT6* were more resistant to 2DG than were cells transformed with plasmid 4H6 that contains only *HXT3*.

### Effect of aneuploidy on gene expression

**Fig 7:**
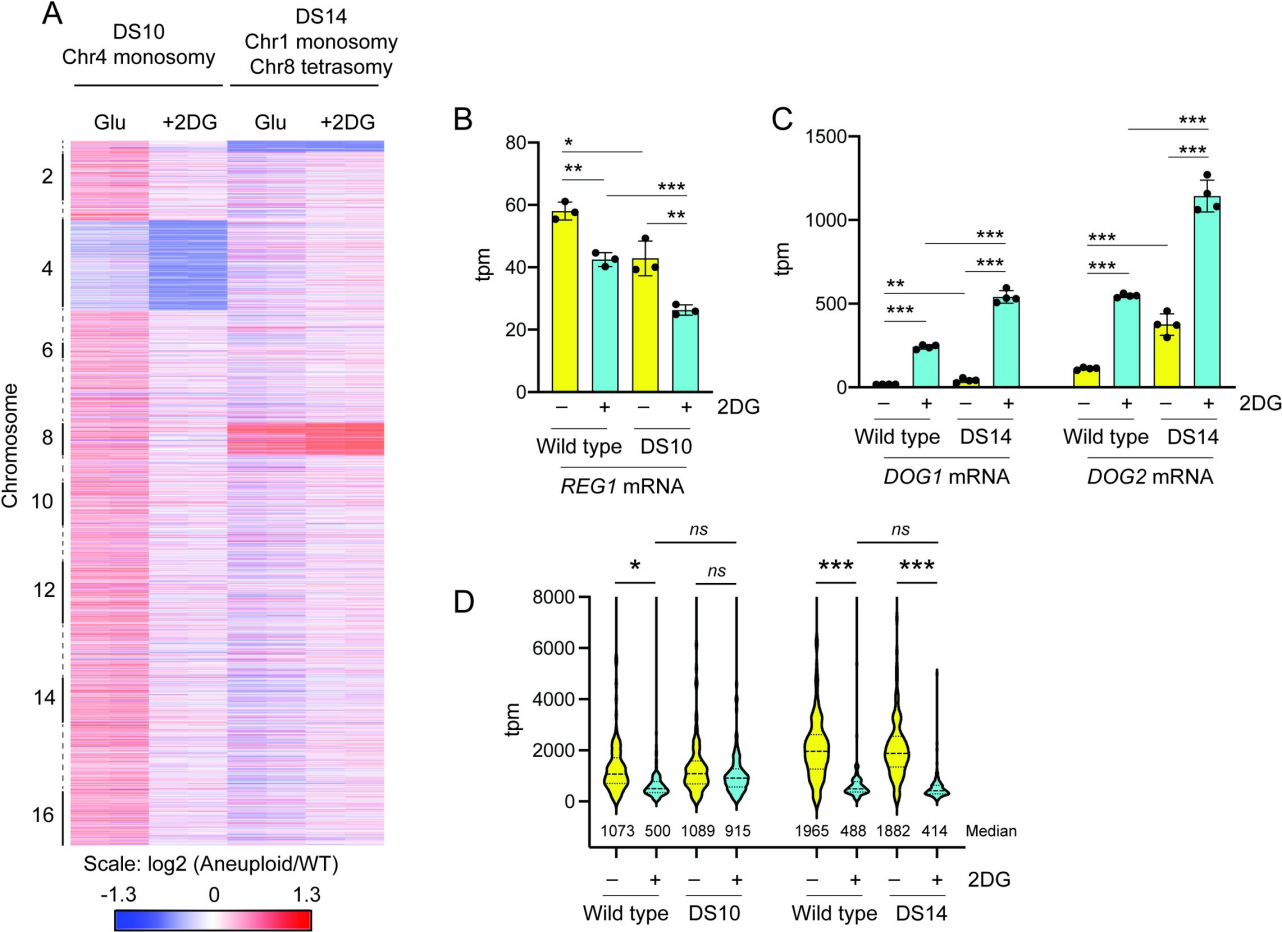
Effect of Aneuploidy on mRNA expression. **A.** Analysis of mRNA abundance was performed using RNAseq in wild type and DS10 cells washed off agar plates and grown for 2 hours in SC media with 2% glucose with and without 2DG. The log2 ratio of mRNA abundance from aneuploid strain divided by the wild type abundance is shown. Genes on the vertical axis are clustered by chromosome. The same RNAseq analysis was also performed on wild type and DS14 cells grown to mid-log in SC media and two hours after addition of 2DG. Two biological replicates for each strain and condition are shown. **B.** *REG1* mRNA abundance as transcripts per million mapped reads (tpm) is shown for wild type and DS10 cells with and without addition of 2DG. **C.** *DOG1* and *DOG2* mRNA abundance is shown for wild type and DS14 cells with and without addition of 2DG. **D.** Ribosomal protein mRNA levels for wild type and aneuploid strains with and without 2DG treatment. TPM Values for 132 mRNAs are shown in a violin plot with the median value indicated below.

To characterize the effect of aneuploidy on gene expression in response to 2DG, we utilized RNAseq to measure mRNA abundance in wild type cells and two aneuploid cells, DS10 and DS14. Because the chromosome 4 monosomy present in DS10 reverts to normal ploidy at high frequency (Fig 4A), RNA was extracted from wild type and DS10 cells washed off agar plates and grown in liquid media for two hours in the presence and absence of 2DG. In contrast, DS14 cells have a stable aneuploid state and RNA was prepared from cells grown to mid-log in liquid media and two hours after addition of 2DG. To visualize the effect of chromosome copy number on mRNA abundance, we generated a heat map in which the color represents the log2 ratio of the mRNA abundance in the aneuploid cell divided by the abundance of the mRNA in the wild type cell. The genes are sorted by chromosome on the vertical axis with the location of individual chromosomes indicated. We found that mRNA abundance is generally proportional to gene copy number (Fig 7A). Genes from the monosomic chromosome 4 in DS10 or chromosome 1 in DS14 showed reduced expression while genes on the tetrasomic chromosome 8 were over-expressed. Analysis of the individual transcript abundance support the idea that reduced expression of the *REG1* gene on chromosome 4 (Fig 7B) and overexpression of the *DOG1* and *DOG2* genes on chromosome 8 (Fig 7C) are important drivers of 2DG resistance in these aneuploid strains.

In 2DG-sensitive haploid cells, ribosomal protein mRNA abundance is greatly reduced following exposure to 2DG [6]. In contrast, 2DG-resistant strains exhibited a much smaller down-regulation of ribosomal protein mRNAs. Wild type diploid strains also show this down-regulation of ribosomal protein mRNA abundance (Fig 7D). The aneuploid cells with the monosomic chromosome 4 showed a dampened response while the cells with the tetraploid chromosome 8 showed a robust down-regulation of ribosomal protein mRNA expression similar to that seen in 2DG-sensitive cells. These data suggest that the chromosome 4 monosomy and chromosome 8 tetrasomy may confer 2DG resistance via distinct mechanisms.

## Discussion

**Fig 8:**
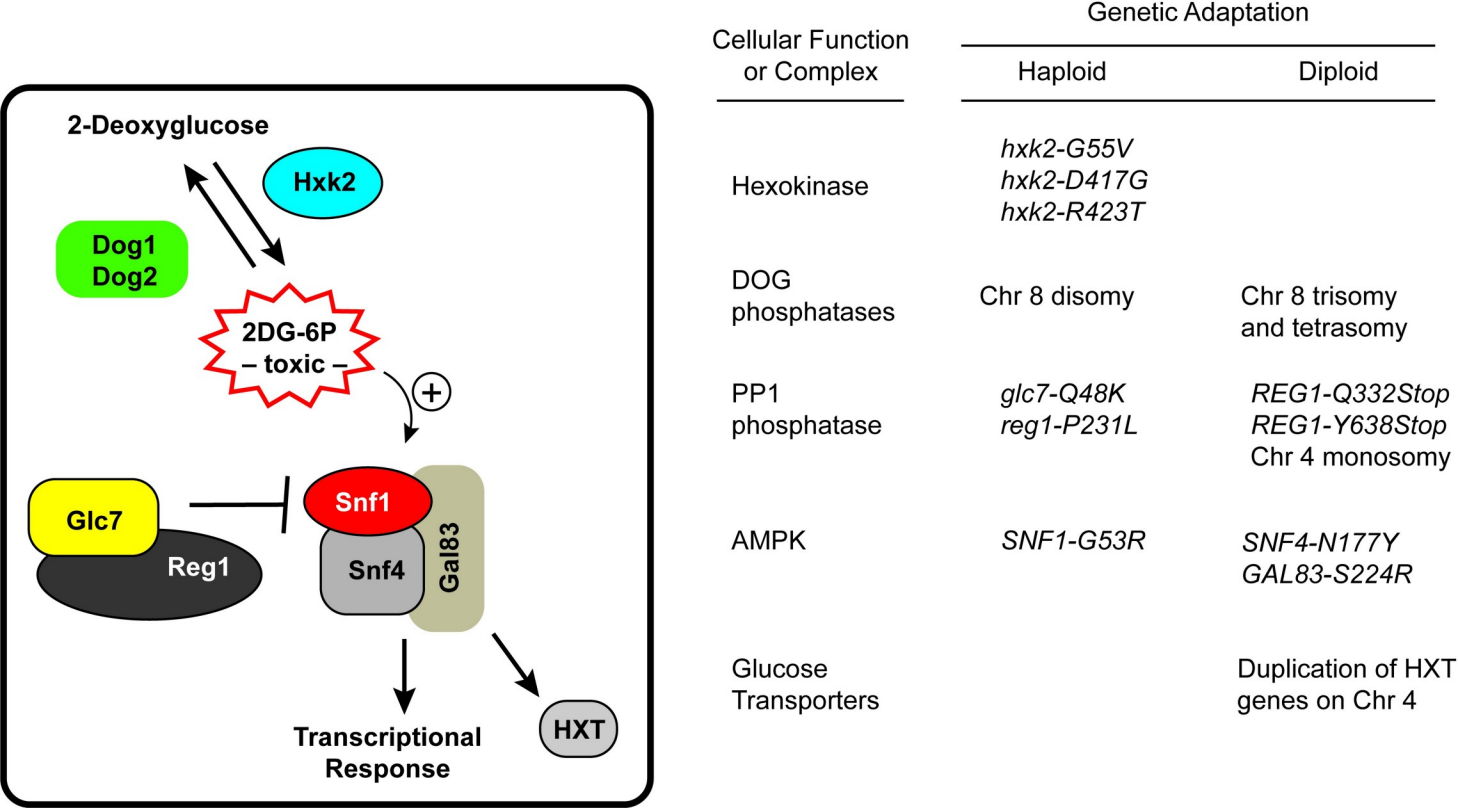
Genetic response to 2DG in haploid and diploid cells. A schematic representation of the yeast cell response to 2DG is shown and described in more detail in the Discussion. Genetic strategies used by haploid and diploid cells to adapt to the presence of 2DG are listed. Recessive alleles are shown in lower case and dominant alleles are shown in upper case.

Two-deoxyglucose is a potent inhibitor of glycolysis in both yeast and mammalian cells growing on glucose [11,25]. Yeast cells adapt to the presence of 2DG by employing two general strategies. First, cells can reduce the accumulation of the toxic metabolite, 2-deoxyglucose-6-phosphate (2DG-6P) or second, cells can activate the AMPK signaling pathway (Fig 8). The idea that 2DG-6P is the toxic metabolite is supported by fact that hexokinase deleted cells (*hxk1Δ hxk2Δ glk1Δ*) lacking the ability to phosphorylate 2DG are completely resistant to 2DG [6]. Also, growth kinetics of cells recovering from 2DG exposure are consistent with the accumulation of a toxic intermediate [11].

In order to reduce the accumulation of 2DG-6P, cells can either reduce its production or increase its degradation. 2DG-6P is produced by hexokinase enzymes and recessive alleles in the *HXK2* gene that reduce the enzyme’s catalytic activity confer resistance to 2DG [6,21]. Why mutations in the *HXK2* gene confer 2DG resistance and not mutations in the other two hexokinase genes in yeast is a puzzle that remains to be solved. Both Hxk1 and Hxk2 enzymes are able to phosphorylate 2DG in vitro [6] yet only mutations in the *HXK2* gene confer 2DG resistance. Loss of function alleles in the *HXK2* gene is a genetic adaptation that has only been observed in haploid cells [5,11,21,26] suggesting that diploid cells heterozygous at the *HXK2* locus do not have a growth advantage in 2DG. In order to increase the rate of 2DG-6P degradation, both haploid and diploid cells increase the expression of the *DOG1* and *DOG2* genes by increasing the copy number of chromosome 8. 2DG resistance exhibits a strong positive correlation with *DOG* gene copy number (Fig 5C). Extra copies of chromosome 8 lead to increased gene expression from almost all the 560 genes on that chromosome (Fig 7A), making this genetic mechanism seem a rather blunt and indiscriminate tool for increasing expression of two genes. Deletion of one of the *DOG* loci from haploid cells with a chromosome 8 disomy essentially eliminates the 2DG resistance (Fig 5D), thus demonstrating that increased expression of these two genes is the key driver for this adaptation to 2DG. One reason that this seemingly blunt tool is a common adaptation to 2DG is that cells with extra copies of chromosome 8 show little or no decrease in fitness under laboratory conditions [27]. We have yet to detect any spontaneous reversion to normal ploidy in cells with extra copies of chromosome 8. The importance of the *DOG* genes and the robust down-regulation of ribosomal protein expression (Fig 7D) suggest that the molecular mechanism of 2DG resistance in cells with extra copies of chromosome 8 is most likely due to the decreased concentration of the toxic 2DG-6-phosphate.

The second general strategy for adaptation to 2DG is to activate the AMPK signaling pathway. AMPK signaling is critical to the adaptation to growth in the presence of 2DG. Cells lacking AMPK (*snf1Δ*) are hypersensitive to 2DG whereas cells with an activated allele of *SNF1* are resistant [11]. AMPK signaling is necessary to maintain expression and plasma membrane retention of hexose transporters in cells undergoing 2DG stress [23]. To achieve activation of this signaling pathway, yeast utilize several distinct genetic adaptations. The most direct mechanism for activation of AMPK is the acquisition of dominant, missense alleles in the genes encoding the AMPK subunits. This strategy was utilized in haploid cells with the *SNF1-G53R* allele [6] and in diploid cells with the *GAL83-S224R* and *SNF4-N177Y* alleles. Thus dominant, missense alleles in the genes coding all three subunits of the AMPK heterotrimer can confer 2DG resistance (Fig 2). Interestingly, these studies highlight the subtle differences in the AMPK isoforms since the *GAL83-S224R* allele can activate AMPK signaling where the analogous missense mutations in the alternative beta subunit genes, *SIP1* and *SIP2*, do not activate their cognate AMPK isoforms.

A second mechanism to activate AMPK signaling is to reduce activity in the PP1 phosphatase, an inhibitor of AMPK activity. The PP1 isoform that dephosphorylates and inactivates yeast AMPK is composed of the catalytic subunit, Glc7, bound to the regulatory subunit, Reg1 [28,29]. The *GLC7* gene is essential thus loss-of-function alleles are not viable and not recovered in genetic screens. The *REG1* gene is not essential; loss-of-function alleles do activate AMPK signaling and confer 2DG resistance however, they come with severe fitness costs [6]. Haploid cells acquire specific missense alleles in both *GLC7* and *REG1* that confer resistance but with much lower fitness costs compared to loss-of-function alleles. In diploid cells, nonsense, loss-of-function alleles in *REG1* confer 2DG resistance through haploinsufficiency (Fig 3). Diploid cells that are heterozygous at the *REG1* locus (*reg1Δ/REG1*) exhibit modest 2DG resistance without the slow growth phenotype in the absence of 2DG observed in diploid cells homozygous for the *reg1Δ* allele.

Diploid cells with a chromosome 4 monosomy are resistant to 2DG (Fig 4B) and show reduced gene expression across the entire chromosome (Fig 7A). The *REG1* gene is on chromosome 4 and we suspect that it is the reduced expression of *REG1* (Fig 7B) that confers 2DG resistance to cells with the chromosome 4 monosomy. However, this chromosome encodes 850 open reading frames and it is possible reduced expression of multiple genes could contribute to 2DG resistance. The greatest experimental challenge to working with cells with a chromosome 4 monosomy is that this aneuploid state is unstable [30] and readily reverts to normal 2n ploidy (Fig 4C). The rate of reversion for the chromosome 4 monosomy (1 in 5 colonies) is much higher than the rate of reversion observed (1 in 10^3^) for disomic states responsible for the phenotypic switching of yeast colony phenotype [8]. We have not been able to genetically manipulate cells with a chromosome 4 monosomy (e.g., by plasmid transformation) without reversion to normal ploidy. However, cells with the chromosome 4 monosomy are easily recognized on agar plates by their small colony phenotype, allowing us to obtain relative pure populations of chromosome 4 monosomy cells for experiments such as RNAseq and whole genome sequencing. Our experiments confirm that this aneuploid state confers 2DG resistance though it comes with a large fitness cost detected as a slow growth rate in the absence of 2DG. Monosomies are not the only aneuploid states that are readily reversible. Haploid yeast cells respond to high pH stress by retaining an extra copy of chromosome 5, an aneuploid state that readily reverts when the pH stress was relaxed [9,31].

One characteristic response to 2DG in wild type cells is the down-regulation of ribosomal protein expression [6]. Strains that have acquired resistance to 2DG, including those with mutations in the PP1 phosphatase, do not exhibit as strong a down-regulation of these genes. Cells with a monosomy of chromosome 4 also show a reduced down-regulation of the ribosomal protein genes (Fig 7D), supporting the idea that reduced expression of *REG1*, a PP1 phosphatase subunit, is a key driver of the chromosome 4 monosomy. The great advantage of this genetic strategy is that the chromosome 4 monosomy is freely reversible. Diploid cells can reap the benefits of a chromosome 4 monosomy when 2DG is present and rapidly return to normal ploidy when the 2DG selection pressure is removed (Fig 4C). The reversibility of the chromosome 4 monosomy is distinct advantage over other genetic strategies, such as dominant alleles and haploinsufficiency, which are not easily reversed.

Downstream targets of AMPK signaling that are important for 2DG resistance are the glucose transporters. Cells challenged with 2DG increase the endocytosis of the glucose transporters in a process that is promoted by the α-arrestins Rod1 and Rog3 [23]. AMPK signaling inhibits these α-arrestins and promotes plasma membrane retention of the glucose transporters. One of the 2DG-resistant diploid strains isolated here contained a duplication of a 117 kb region of chromosome 4 (Fig 6). This duplication, which included 3 *HXT* genes, conferred 2DG resistance and we showed that over-expression of the *HXT3* gene was sufficient for 2DG resistance. Rather than activating AMPK and affecting all downstream targets, this genetic adaptation affects one specific target of the AMPK signaling. The termini of the duplicated region mapped to retrotransposons, suggesting a nonequal homologous recombination event could have generated this duplication in one cell with the corresponding deletion in the other cell (not viable in haploid cells due to loss of several essential genes). This mechanism for increasing gene expression using homologous retrotransposon sequences has been documented in other studies [32] but would not be possible for the *DOG* genes since the retrotransposons flanking the *DOG* genes would encompass the centromere on chromosome 8. If this region were duplicated, a dicentric and unstable chromosome would have been generated.

Using selection for 2DG resistance as a model for the selection of drug resistance, our studies show that numerous genetic adaptations are utilized to impact a relatively simple pathway of drug resistance (Fig 8). Both *Saccharomyces* cells grown in the laboratory and clinical isolates of *Candida* from human patients utilize aneuploidy as a means for acquisition of drug resistance [33]. Human cancer cells also make wide use of aneuploidy and chromosomal rearrangements as they respond to changing tumor environments and selective pressures [34]. Our studies suggest that while aneuploidy may be a blunt tool for altering gene expression, the potential for reversibility of aneuploid states make them a particularly useful mechanism for rapidly responding to changing selective pressures.

## Materials and methods

### Yeast strains and growth conditions

The yeast strains used in this study were all derivatives of S288C. Yeast strains with specific gene deletions were generated in our laboratory or by the *Saccharomyces* Genome Deletion Project [35] and purchased from Thermo Fisher Scientific (Table 1). Cells were grown at 30°C using standard synthetic complete media lacking nutrients needed for plasmid selection [36].

### Selection of Spontaneous 2DG-resistant mutants

Spontaneous mutations that conferred 2DG resistance were selected in the diploid strain MSY1527 (Table 1). Approximately 2x10^7^ cells were spread on agar plates containing synthetic complete media with 2% glucose (g/100ml) and 0.1% 2-deoxyglucose. Plates were incubated at 30°C for 4–6 days, and 2DG-resistant colonies were isolated for further study.

### Whole genome sequencing

The genomes of the 2DG-resistant strains were analyzed by whole genome sequencing. Genomic DNA was prepared using a glass bead phenol extraction method [37]. Sequencing libraries were prepared using a modified Illumina Nextera protocol and multiplexed onto a single run on an Illumina NextSeq500 to produce 151-bp paired-end reads [38]. Sequencing produced an average depth of 10–20 million reads per sample. Reads were mapped against the reference genome of strain S288C using Bowtie2 and SAMtools [39,40]. Variants were detected using VFCTools [41]. Read depth along each chromosome was measured using BEDTools [42]. Chromosome ploidy was determined by plotting the normalized median read depth for each chromosome.

### Mutagenesis and plasmid construction

Oligonucleotide-directed mutagenesis of plasmids was performed with *Pfu* polymerase, followed by digestion of the starting plasmid template with the restriction enzyme *DpnI* [43]. Mutagenesis was confirmed by DNA sequencing the entire open reading frames to insure successful mutagenesis with no unintended sequence alterations. The *DOG1/2* plasmids were made by inserting the 4.1 kb BamHI—EcoRI fragment (chromosome 8; 192,277 to 196,376) into pRS316 (low copy number) and pRS426 (high copy number) plasmids [44].

### 2-deoxyglucose resistance assays

2DG resistance was measured in synthetic complete media with 2% glucose (g/100ml) as previously described in McCartney, Chandrashekarappa (11) using either a titration of increasing concentration of 2DG or multiple replicates of cultures grown from independent colonies with and without 0.1% 2DG.

### RNAseq analysis

RNA samples were prepared from multiple independent yeast cultures grown on synthetic complete medium using the RNeasy Mini Kit (Qiagen). Sequencing libraries were prepared using the TruSeq Stranded mRNA library method (Illumina). RNA sequences were mapped to *S*. *cerevisiae* mRNA using the kallisto software package [45]. Each RNA sample yielded 40–50 million reads. mRNA abundance was expressed in transcripts per million mapped reads (tpm). Comparison of mRNA expression under different conditions utilized a Student’s t-test to calculate a p values with a false discovery rate threshold of 0.01%. Read depth was determined using Bowtie2, SAMTools and BEDTools [39,40,42]. RNA expression was visualized using a heat map generated by Morpheus software developed at the Broad Institute of Cambridge Massachusetts.

### Statistical significance

Unless otherwise stated, mean values were calculated from a minimum of three independent measurements, and the error bars represent 1 standard deviation. Statistical significance was determined using the Student t test for unpaired variables with equal variance. In all cases, *p* values are indicated as follows: * p<0.05; ** p<0.01; *** p<0.001.

## Supporting information

S1 Fig Alignment and structure of the AMPK beta subunit carbohydrate binding modules. (A) Multiple sequence alignment of the yeast and human (Hs) beta subunit CBM domains. Position of the seven beta sheets (S1-S7) are indicated. The serine residue corresponding to Gal83-S224, Sip2-S226 and Sip1-S541 is indicated by the asterisks. (B) Three dimensional structure of the Sip2 CBM showing the seven beta sheets and the position of S226 (red mesh) is shown using the structure coordinates in 2QLV.pdb [47]. (C and D) 2DG resistance assays performed in quadruplicate using wild type (WT) and gene deletion strains transformed with the indicated plasmids that express a wild type gene, the same gene with a single missense mutation indicated or empty plasmid vector. Mean values statistically different from cells expressing the wild type allele are indicated.
(TIF)

S2 Fig Disomy of chromosome 8 is associated with 2DG resistance. Haploid strains RS17, RS18 and RS19 with multiple disomic chromosomes were mated with wild type haploid of opposite mating type (MSY188). After sporulation, the haploid progeny were analyzed for 2DG resistance by replica plating and scored as resistant (R) or sensitive (s). Four tetrads from each cross that showed 2:2 segregation of 2DG resistance were selected and the pooled DNA from resistant and sensitive strains was sequenced. The normalized median read depth for each chromosome of the parents (MSY188, RS17, RS18 and RS19) is plotted along with the resistant and sensitive pools.
(TIF)

S3 Fig Deletion of *DOG1/2* and *HXT415* genes in cells with disomy of chromosome 8. **(**A) Distribution of whole genome sequence read depth for each chromosome is plotted for four haploid strains with median read depth for each chromosome indicated by the yellow bar. (B) Read depth is plotted as a function of position along chromosome 8 near the *DOG1/2* locus for MSY1553 (black) and MSY1560 (blue). The gene map for this region of chromosome 8 is shown above. (C) Distribution of read depth 10 kb before, 10 kb after and across the *DOG1/2* locus is plotted for MSY1553 (black) and MSY1560 (blue). The median value is indicated by the yellow bar in the plot and numerically below. (D) Read depth is plotted as a function of position along chromosome 8 near the *HXT415* locus for MSY1553 (black) and MSY1562 (blue). The gene map for this region of chromosome 8 is shown above. (E) Distribution of read depth 10 kb before, 10 kb after and across the *HXT415* locus is plotted for MSY1553 (black) and MSY1562 (blue). The median value is indicated by the yellow bar in the plot and numerically below.
(TIF)

S1 Table RNAseq data for wild type and aneuploid strains. Transcripts per million mapped reads (tpm) is provided for 5917 yeast open reading frames. Samples S72-S83 are triplicate datasets from wild type diploid cells (MSY1527) and DS10 cells (chromosome 4 monosomy) washed off plates and grown for two hours in SC media with 2% glucose or in the same media with 0.1% 2DG. Samples S132-S147 are quadruplicate datasets from wild type diploid cells (MSY1527) and DS14 cells (chromosome 1 monosomy and chromosome 8 tetrasomy) grown to mid-log in SC media with 2% glucose and two hours after addition of 2DG to 0.1%.
(XLSX)
