# Supplementary figures and images for: Drug resistance in diploid yeast is acquired through dominant alleles, haploinsufficiency, gene duplication and aneuploidy
Source: PLoS Genet. 2021 Sep 23;17(9):e1009800. doi: 10.1371/journal.pgen.1009800 (PMC8460028; doi:10.1371/journal.pgen.1009800)

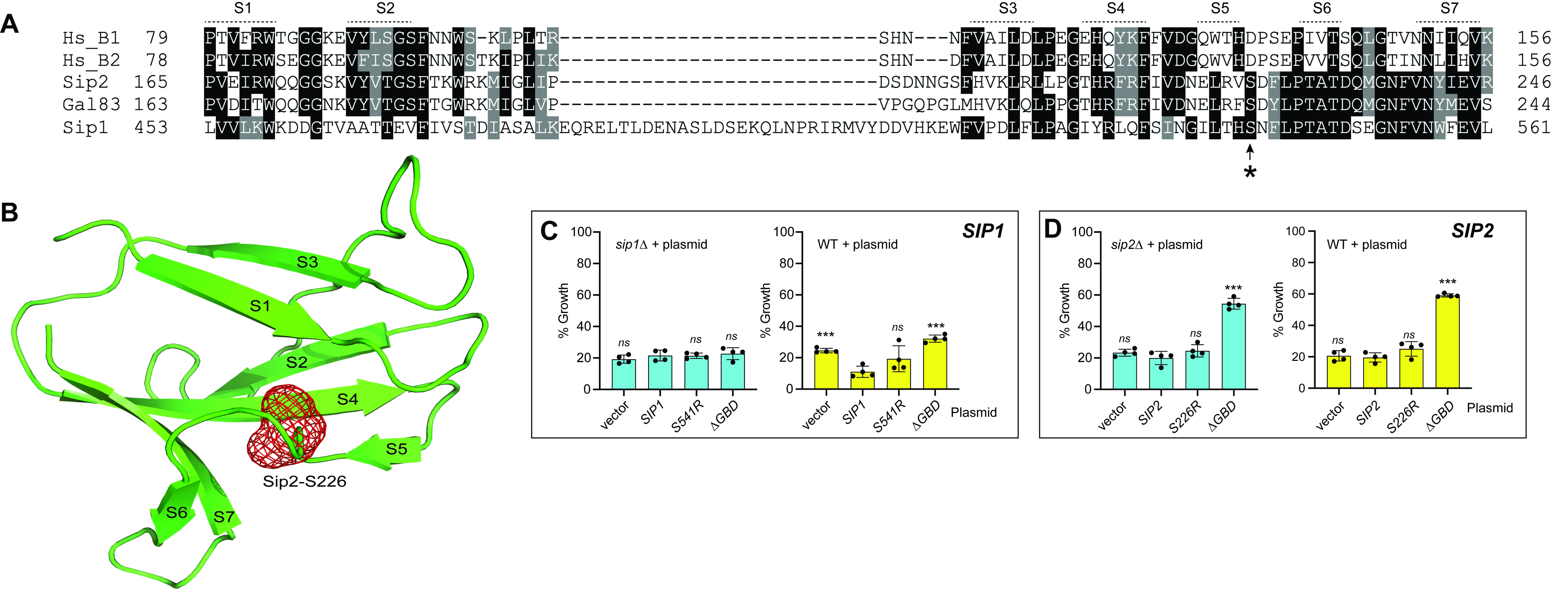

Supplement: S1 Fig — (A) Multiple sequence alignment of the yeast and human (Hs) beta subunit CBM domains. Position of the seven beta sheets (S1-S7) are indicated. The serine residue corresponding to Gal83-S224, Sip2-S226 and Sip1-S541 is indicated by the asterisks. (B) Three dimensional structure of the Sip2 CBM showing the seven beta sheets and the position of S226 (red mesh) is shown using the structure coordinates in 2QLV.pdb [47]. (C and D) 2DG resistance assays performed in quadruplicate using wild type (WT) and gene deletion strains transformed with the indicated plasmids that express a wild type gene, the same gene with a single missense mutation indicated or empty plasmid vector. Mean values statistically different from cells expressing the wild type allele are indicated. (TIF) [file pgen.1009800.s001.tif]

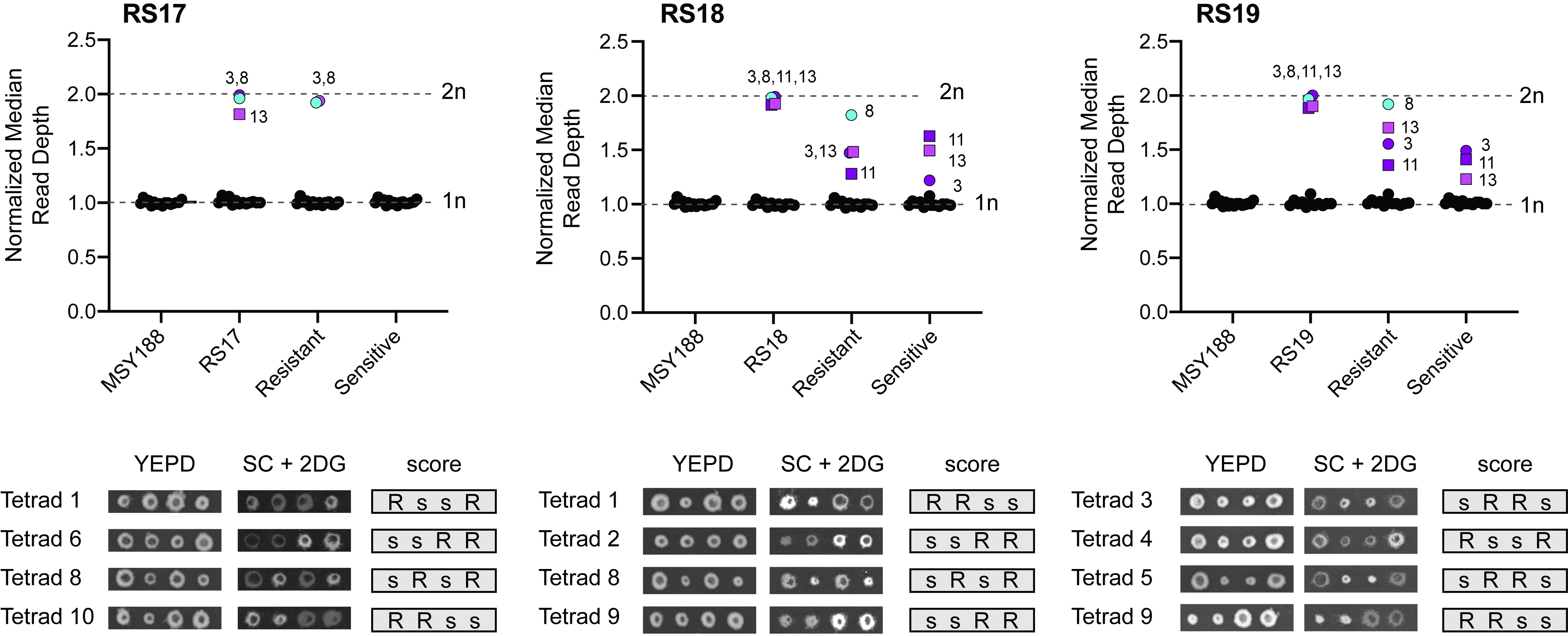

Supplement: S2 Fig — Haploid strains RS17, RS18 and RS19 with multiple disomic chromosomes were mated with wild type haploid of opposite mating type (MSY188). After sporulation, the haploid progeny were analyzed for 2DG resistance by replica plating and scored as resistant (R) or sensitive (s). Four tetrads from each cross that showed 2:2 segregation of 2DG resistance were selected and the pooled DNA from resistant and sensitive strains was sequenced. The normalized median read depth for each chromosome of the parents (MSY188, RS17, RS18 and RS19) is plotted along with the resistant and sensitive pools. (TIF) [file pgen.1009800.s002.tif]

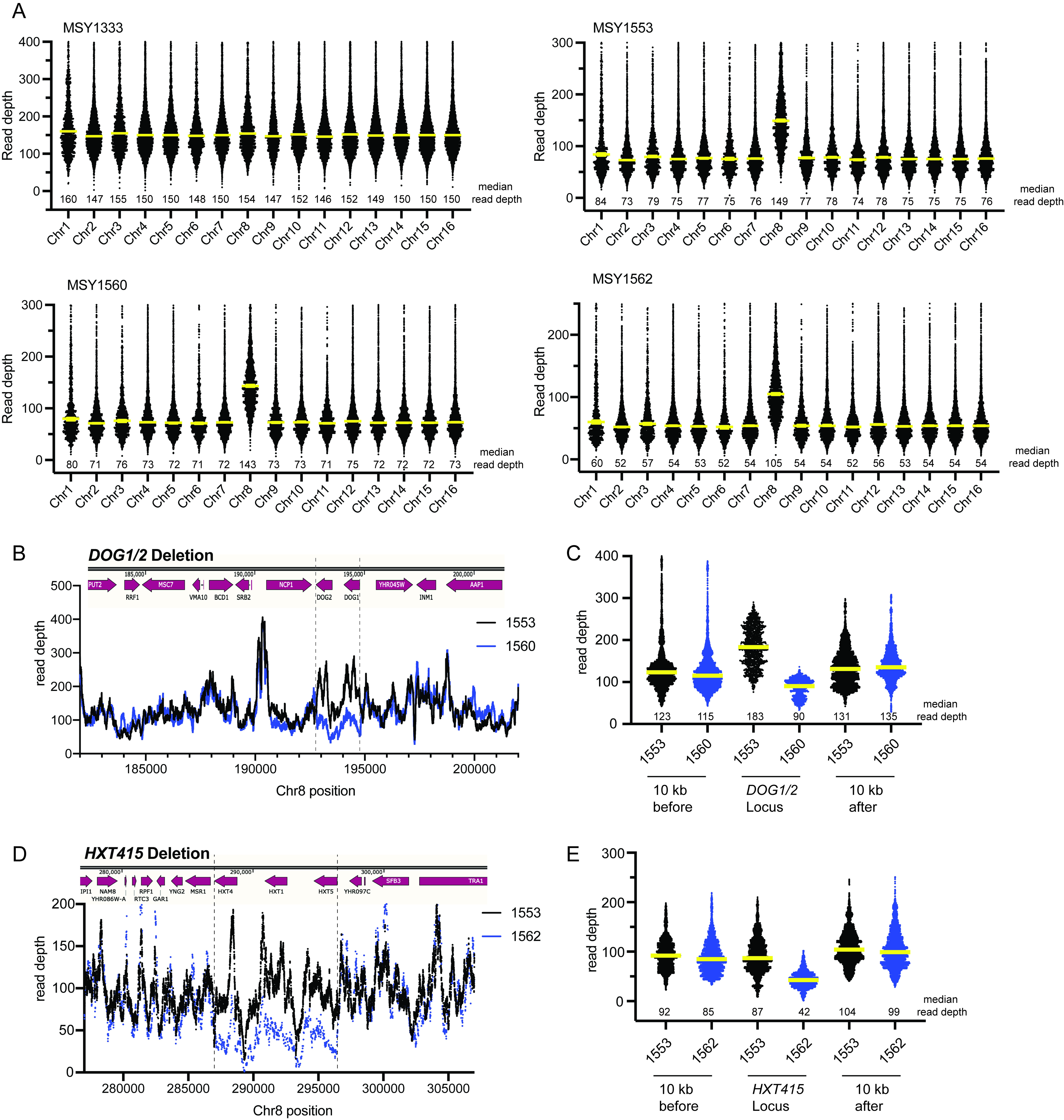

Supplement: S3 Fig — (A) Distribution of whole genome sequence read depth for each chromosome is plotted for four haploid strains with median read depth for each chromosome indicated by the yellow bar. (B) Read depth is plotted as a function of position along chromosome 8 near the DOG1/2 locus for MSY1553 (black) and MSY1560 (blue). The gene map for this region of chromosome 8 is shown above. (C) Distribution of read depth 10 kb before, 10 kb after and across the DOG1/2 locus is plotted for MSY1553 (black) and MSY1560 (blue). The median value is indicated by the yellow bar in the plot and numerically below. (D) Read depth is plotted as a function of position along chromosome 8 near the HXT415 locus for MSY1553 (black) and MSY1562 (blue). The gene map for this region of chromosome 8 is shown above. (E) Distribution of read depth 10 kb before, 10 kb after and across the HXT415 locus is plotted for MSY1553 (black) and MSY1562 (blue). The median value is indicated by the yellow bar in the plot and numerically below. (TIF) [file pgen.1009800.s003.tif]
